# Orientation and Contrast Tuning Properties and Temporal Flicker Fusion Characteristics of Primate Superior Colliculus Neurons

**DOI:** 10.3389/fncir.2018.00058

**Published:** 2018-07-24

**Authors:** Chih-Yang Chen, Ziad M. Hafed

**Affiliations:** ^1^Physiology of Active Vision Laboratory, Werner Reichardt Centre for Integrative Neuroscience, Tübingen University, Tübingen, Germany; ^2^Graduate School of Neural and Behavioural Sciences, International Max Planck Research School, Tübingen University, Tübingen, Germany; ^3^Department of Cognitive Neurology, Hertie Institute for Clinical Brain Research, Tübingen University, Tübingen, Germany

**Keywords:** superior colliculus, orientation tuning, contrast sensitivity, center-surround interactions, temporal frequency tuning, flicker fusion

## Abstract

The primate superior colliculus is traditionally studied from the perspectives of gaze control, target selection, and selective attention. However, this structure is also visually responsive, and it is the primary visual structure in several species. Thus, understanding the visual tuning properties of the primate superior colliculus is important, especially given that the superior colliculus is part of an alternative visual pathway running in parallel to the predominant geniculo-cortical pathway. In recent previous studies, we have characterized receptive field organization and spatial frequency tuning properties in the primate (rhesus macaque) superior colliculus. Here, we explored additional aspects like orientation tuning, putative center-surround interactions, and temporal frequency tuning characteristics of visually-responsive superior colliculus neurons. We found that orientation tuning exists in the primate superior colliculus, but that such tuning is relatively moderate in strength. We also used stimuli of different sizes to explore contrast sensitivity and center-surround interactions. We found that stimulus size within a visual receptive field primarily affects the slope of contrast sensitivity curves without altering maximal firing rate. Additionally, sustained firing rates, long after stimulus onset, strongly depend on stimulus size, and this is also reflected in local field potentials. This suggests the presence of inhibitory interactions within and around classical receptive fields. Finally, primate superior colliculus neurons exhibit temporal frequency tuning for frequencies lower than 30 Hz, with critical flicker fusion frequencies of <20 Hz. These results support the hypothesis that the primate superior colliculus might contribute to visual performance, likely by mediating coarse, but rapid, object detection and identification capabilities for the purpose of facilitating or inhibiting orienting responses. Such mediation may be particularly amplified in blindsight subjects who lose portions of their primary visual cortex and therefore rely on alternative visual pathways including the pathway through the superior colliculus.

## Introduction

It has been shown since more than 70 years ago that superior colliculus (SC) neurons have visual responses. Almost in parallel with these findings, researchers have recognized that the SC is also a crucial midbrain structure for orienting behavior, especially for saccadic eye movements in primates (Wurtz and Optican, 1994). As a result, there was a relative abundance of studies in the primate model aimed at understanding subcortical connections for movement control, as well as cortical neural substrates for eye movements (Gandhi and Katnani, 2011). More recently, studies on the role of the primate SC in active vision have focused more heavily on cognitive tasks like target selection and visual attention (Krauzlis et al., 2013; Basso and May, 2017; Crapse et al., 2018; Odegaard et al., 2018). The visual properties of primate SC neurons, on the other hand, have been largely left out after some simple characterizations using light dots and bars.

In separate lines of research, it became recognized that saccadic viewing patterns, orienting efficiency, and target selection can drastically differ under a variety of visual conditions and in natural scene scenarios, and depending on factors like stimulus salience (White et al., 2008, 2017a,b; Veale et al., 2017). This means that low level image statistics can strongly influence eye movements (Ludwig et al., 2004; White et al., 2008; Chen and Hafed, 2017). Motivated by this, we have recently begun to characterize primate SC visual properties from an ecological perspective (Hafed and Chen, 2016). Our starting point was that the primate SC can be an important neural substrate for implementing visual salience maps that can guide behavior (Veale et al., 2017; White et al., 2017a,b) and this is consistent with how visual topography in the SC seems to be already co-registered with the deeper saccade map topography in the same structure (Cynader and Berman, 1972; Robinson, 1972). We have shown that visual (and saccade-related) topography is asymmetric between upper and lower visual fields (Hafed and Chen, 2016) and also that spatial frequency tuning properties of SC neurons allow this structure to facilitate gaze behavior under natural scene scenarios in which low spatial frequencies are predominant (Hafed and Chen, 2016; Chen and Hafed, 2017; Chen et al., in press). Here, we continued our investigation of the primate SC's visual properties. We focused on orientation tuning, especially given that recent rodent work has yielded some controversy (Ahmadlou and Heimel, 2015; Feinberg and Meister, 2015; Inayat et al., 2015). We also explored potential center-surround RF interactions as well as temporal frequency tuning properties.

Our efforts in the present paper, along with those in our recently published manuscripts, are specifically aimed at complementing early seminal work on the visual functions of the primate SC, primarily performed in the 1970's. For example, Schiller and Koerner conducted experiments on awake primates, like in our case, and reported no direction or orientation selectivity, although this was not explicitly quantified (Schiller and Koerner, 1971). Goldberg and Wurtz also studied awake animals and reported 10% of neurons having directional selectivity, but these authors did not examine orientation selectivity (Goldberg and Wurtz, 1972). We aimed to quantify such selectivity in the present study. Both groups of authors also described atypical center-surround RF interactions (Schiller and Koerner, 1971; Goldberg and Wurtz, 1972) but there were no investigations of how factors like stimulus contrast, sustained visual responses, and local population activity were related to these interactions; a primary objective of our current study was exactly to examine such factors. Finally, Schiller and Koerner also described the SC as a jerk detector (Schiller and Koerner, 1971) but response sensitivity to specific temporal frequencies of stimuli was not mapped, as we have done here. More broadly, the great majority of remaining early work on the visual functions of the primate SC has been performed on anesthetized animals as opposed to awake ones. For example, similar descriptions to those in Schiller and Koerner (1971) were made for anesthetized monkeys in Cynader and Berman (1972), Marrocco and Li (1977) and Moors and Vendrik (1979). Anesthetized monkeys were also used to study interactions of relative motion within SC RF's (Davidson and Bender, 1991). We believe that revisiting such early work (from both anesthetized and awake monkeys) with a more modern context is critical looking forward from now, especially for fully understanding how the SC can support perception, cognition, and action under a variety of naturalistic conditions, including social interactions (Chen et al., in press).

In all, we believe that our experiments, coupled with our recent findings on visual field topography, contrast sensitivity, and spatial frequency tuning (Chen et al., 2015, in press; Hafed and Chen, 2016; Chen and Hafed, 2017) support hypotheses that the SC can play an important role in determining visual capabilities during rapid orienting behavior, and also during clinical conditions of blindsight, in which loss of conscious visual perception through loss of primary visual cortex (V1) results in residual visual capabilities that can reflect involvement of alternative visual pathways (Yoshida et al., 2008, 2017; Cowey, 2010; Kato et al., 2011; Takaura et al., 2011; Leopold, 2012).

## Materials and methods

Our monkey experiments were approved by the regional governmental offices in the city of Tuebingen (Regierungspräsidium Tübingen). The study was conducted in strict compliance of the European Union guidelines on animal research, as well as the associated German laws enforcing these guidelines.

### Animal preparation

Rhesus macaque monkeys P and N (male, *Macaca mulatta*, aged 7 years) were prepared for behavior and superior colliculus (SC) recordings earlier (Chen and Hafed, 2013; Chen et al., 2015). Briefly, we placed a recording chamber centered on the midline and aimed at a stereotaxically defined point 1 mm posterior of and 15 mm above the inter-aural line. The chamber was tilted posterior of vertical (by 38 and 35° for monkeys P and N, respectively). We implanted one eye of each monkey with scleral search coils for tracking eye movements, using high spatial and temporal precision, with the magnetic induction technique (Fuchs and Robinson, 1966; Judge et al., 1980). All implant surgeries (head holders, chambers, and eye coils) were performed under full isoflurane anesthesia, and the animals received a combination of analgesics for several days after each procedure. No experiments were conducted except after full recovery from the surgeries. The animals also benefitted from regular visits from our university's veterinary service, occurring at least once per week.

### Orientation tuning task

We asked the monkeys to perform a pure fixation task while we recorded the activity of visually-responsive SC neurons. In each trial, a white fixation spot (8.5 × 8.5 min arc) appeared over a gray background. Fixation spot and background luminance were described earlier (Chen and Hafed, 2013). After an initial fixation interval (400–550 ms), the fixation spot transiently dimmed for ~50 ms. This was useful for resetting microsaccadic rhythms (Hafed and Ignashchenkova, 2013; Tian et al., 2016, 2018; Bellet et al., 2017). After an additional 110–320 ms, a stationary Gabor patch with 80% relative contrast (defined as Lmax – Lmin/Lmax + Lmin) appeared for ~200 ms within the neuron's response field (RF). The Gabor patch was similar to that described in (Hafed and Chen, 2016; Chen and Hafed, 2017). The RF was estimated earlier in the session using standard saccade tasks (Chen et al., 2015; Hafed and Chen, 2016). In particular, delayed visually-guided and memory-guided saccades were used to allow us to isolate epochs of visual responsiveness (after target onset) or saccade-related discharge (near saccade onset), and we estimate visual and movement RF's by varying the location to which the saccades were made. The Gabor patch size that we presented during a given session (i.e., for a given neuron) was chosen to fill as much of the visual RF (characterized from the saccade tasks) as possible. The spatial frequency of the grating that we used was 2.22 cycles/° (cpd) because this spatial frequency drove our neurons well (Chen et al., 2015). Moreover, the orientation of the grating was varied randomly across trials. In monkey N, the orientations were 0, 30, 60, 90, 120, or 150° clockwise from vertical; in monkey P, the orientations were 0, 45, 90, or 135° clockwise from vertical. Grating phase was randomized from trial to trial, and the monkeys were rewarded only for maintaining fixation; no saccadic orienting to the grating or any other behavioral response was required.

We recorded from 43 well-isolated neurons (isolated and characterized online within each experimental session). The range of preferred eccentricities that we sampled (also for our other tasks below) was similar to our earlier studies (Chen et al., 2015; Hafed and Chen, 2016); also see **Figure 5E**. We excluded trials with microsaccades occurring within ±100 ms from stimulus onset because such occurrence can alter neural activity (Hafed, 2011; Hafed et al., 2015). Because of this, we further excluded 12 neurons that we had recorded, since they did not have >20 repetitions for all of the tested orientations. For the neurons that we included here, we had sufficient trials for analysis (we collected >255 trials per neuron; average: 389 ± 175 s.d.).

### Contrast sensitivity task with different stimulus sizes

We used the same contrast sensitivity task of Chen et al. (2015). However, in some trials, the stimulus was filling as much of the RF as possible (referred to here as the “big” stimulus condition), as in Chen et al. (2015) and in other trials, the stimulus was small (0.5–1° in size). This size was chosen to still allow at least 1 cycle of the 2.22 cpd grating to appear within the stimulus. We compared contrast sensitivity curves for the big and small stimuli.

We recorded from 27 neurons, each being exposed to the big (Chen et al., 2015) or small stimuli in the same session. The neurons responses to the big stimuli were included in Chen et al. (2015) but for analyses that were distinct from those that we are focusing on in the present study. In the present study, we compared responses to those with small stimuli (not described in our earlier study). We also excluded trials with microsaccades occurring within ±100 ms from stimulus onset as discussed above. We further excluded 3 recorded neurons since they did not have >25 repetitions for all the tested stimuli. Across neurons, we collected >181 trials per neuron (average: 253 ± 50 s.d.).

### Temporal flicker task

In this task, a vertical Gabor grating of 2.22 cpd spatial frequency (similar to the gratings used in the orientation tuning task above) was flickered within the RF of a neuron for 2,000 ms. Flicker frequency could be 3, 5, 10, 20, or 60 Hz from trial to trial, and the flicker itself was achieved by turning the grating off (i.e., zero contrast grating) at regular temporal intervals dictated by the frequency chosen for any given trial (we used a 50% duty cycle for the flicker). Our display's refresh rate was 120 Hz. To avoid onset and offset transients, we gradually increased stimulus contrast at trial onset in the first 1,000 ms of a trial (to reach 80% contrast), and we similarly gradually decreased stimulus contrast at trial end for the final 1,000 ms of a trial. This was similar to the approach used to study flicker perception capabilities in blindsight human patients (Trevethan and Sahraie, 2003). The monkey was required to maintain fixation throughout the entire stimulus presentation sequence.

We recorded from 55 neurons. Ten neurons were excluded because they did not have >25 repetitions for all the tested stimuli. Across neurons, we collected >137 trials per neuron (average: 166 ± 29 s.d.).

### Neuron classification

We used similar neuron classification criteria to those used in our recent studies (Chen et al., 2015; Hafed and Chen, 2016; Chen and Hafed, 2017). Briefly, a neuron was labeled as visual if its activity 0–200 ms after target onset in a delayed saccade task (Hafed and Krauzlis, 2008; Hafed and Chen, 2016) was higher than activity 0–200 ms before target onset (*p* < 0.05, paired *t*-test). The neuron was labeled as visual-motor if its pre-saccadic activity (−50 to 0 ms from saccade onset) was also elevated in the delayed saccade task relative to an earlier fixation interval (100–175 ms before saccade onset) (Li and Basso, 2008). Our results were similar for either visual or visual-motor neurons, so we combined neuron types in all of our analyses.

### Firing rate analyses

We analyzed SC visual bursts by measuring peak firing rate 20–150 ms after stimulus onset (Chen and Hafed, 2017). We then obtained contrast sensitivity curves as in Chen et al. (2015). Specifically, we estimated semi-saturation contrast, baseline activity, and maximal firing rate (for the highest possible contrast), and we did so for either small or large stimuli. We did so by fitting the measured firing rate data to the following equation:

(1) $$\begin{array}{l} f\left(c\right)=R*\frac{c^{n}}{{C_{50}}^{n}+c^{n}}+B \end{array}$$

where c is the tested contrast (5, 10, 20, 40, 80%) R is the maximal firing rate, C_50_ is the semi-saturation contrast, n is an exponent determining the steepness of the contrast sensitivity curve, and B is the baseline firing rate (obtained from all trials in the interval 0–50 ms before grating onset). The goodness of fit for the above equation was validated according to the approach in Carandini et al. (1997). All of our neurons had >80% explained variance by the fit. To combine different neurons in summary analyses, we first normalized the visual burst activity in each neuron to that observed for the highest contrast, and we then fit the normalized firing rate of all the neurons as one single population (**Figure 2D**). The sustained visual response of neurons was measured by obtaining the mean firing rate 150–250 ms after stimulus onset (**Figure 3D**).

For orientation tuning, we computed an orientation selectivity index (OSI) similar to that used in cortical visual areas, for example (Hansel and van Vreeswijk, 2012). This allowed us to directly compare orientation tuning properties in the SC to other cortical visual areas. Briefly, the OSI was calculated as follows:

(2) $$\begin{array}{l} OSI=\frac{R_{pref}\;-\;{\;R}_{ortho}}{R_{pref}\;+{\;R}_{ortho}} \end{array}$$

where *R*_*pref*_ is the visual burst strength of the neuron for the preferred orientation (i.e., the orientation eliciting the most spikes) and *R*_*ortho*_ is the visual burst strength of the neuron for the orthogonal orientation relative to the preferred one.

To estimate how fast a given visual response was evoked for a given visual condition, we estimated first-spike latency of a visual burst (i.e., the average time from stimulus onset to the first stimulus-evoked action potential by a putative neuron). We did this by using Poisson spike train analysis (Legendy and Salcman, 1985).

For temporal flicker, in addition to raw plots of firing rates, we measured mean firing rate throughout a trial. We then measured all means across trials and plotted the average of these measurements as a function of the temporal frequency of the stimulus. Adopting a similar technique to (Derrington and Lennie, 1984), we fitted these measurements to a function that gave us a “tuning curve” for temporal frequency. The specific function that we used for our data was a difference-of-Gaussians function as follows:

(3) $$\begin{array}{l} f\left(x\right)=a_{1}*e^{-{\left(\frac{x-b_{1}}{c_{1}}\right)}^{2}}-a_{2}*e^{-{\left(\frac{x-b_{2}}{c_{2}}\right)}^{2}}+B \end{array}$$

where *x* is the tested temporal frequency, *a*_1_ and *a*_2_ represent the amplitudes of each Gaussian function, b_1_ and b_2_ represent the means of each Gaussian function, *c*_1_ and *c*_2_ are the bandwidths of each Gaussian function, and B is the baseline firing rate (obtained from all trials in the interval 0–50 ms before grating onset). The goodness of fit was again validated using the approach in Carandini et al. (1997). All of our neurons had >80% explained variance by the fit.

We also performed Fourier transforms on the average firing rates obtained from a given flicker frequency. This allowed us to identify the firing rate amplitude at the primary oscillation frequency of neural fluctuations (F1), which should match the flicker frequency of the stimulus. We then computed the ratio of the amplitude of the oscillation at F1 to the amplitude at 0 Hz (i.e., the mean DC value of the firing rate; called F0) to obtain a sensitivity to the flicker at F1. We computed this sensitivity ratio (called F1/F0) for each tested temporal frequency, and we then fitted the data with a simple exponential decay function where the x-axis is temporal frequency and the y-axis is F1/F0. The stimulus flicker frequency for which the ratio of F1 amplitude to mean firing rate (F1/F0) was below 20% of the peak ratio was taken as the critical flicker fusion frequency of a given neuron (Wells et al., 2001); similarly, the stimulus flicker frequency for which the ratio of F1 firing rate to mean firing rate (F1/F0) was maximal was called the “peak” flicker frequency. For our estimates of preferred frequency, peak F1/F0, and critical flicker frequency, we had a lower and upper bound of 3 and 60 Hz, respectively, because these were the bounds of our sampling range for estimating temporal frequency tuning (e.g., see **Figure 5**).

### Local field potential analyses

For the stimulus size manipulation, we also analyzed local field potentials (LFP's). We obtained LFP's from wide-band neural signals using methods that we described recently (Hafed and Chen, 2016; Chen and Hafed, 2017). We then aligned LFP traces on stimulus onset, and we measured evoked and sustained responses. First, we measured the strongest deflection occurring in the interval 20–150 ms after stimulus onset, to obtain a measure that we called the transient LFP response. Second, we measured the mean deflection in the period 150–250 ms after stimulus onset, to obtain what we referred to as the sustained LFP response. Since the LFP evoked response is negative going, when we refer to a “peak” LFP response, we mean the most negative value of the measured signal.

## Results

### Orientation selective neurons in the primate SC

**Figure 1:**
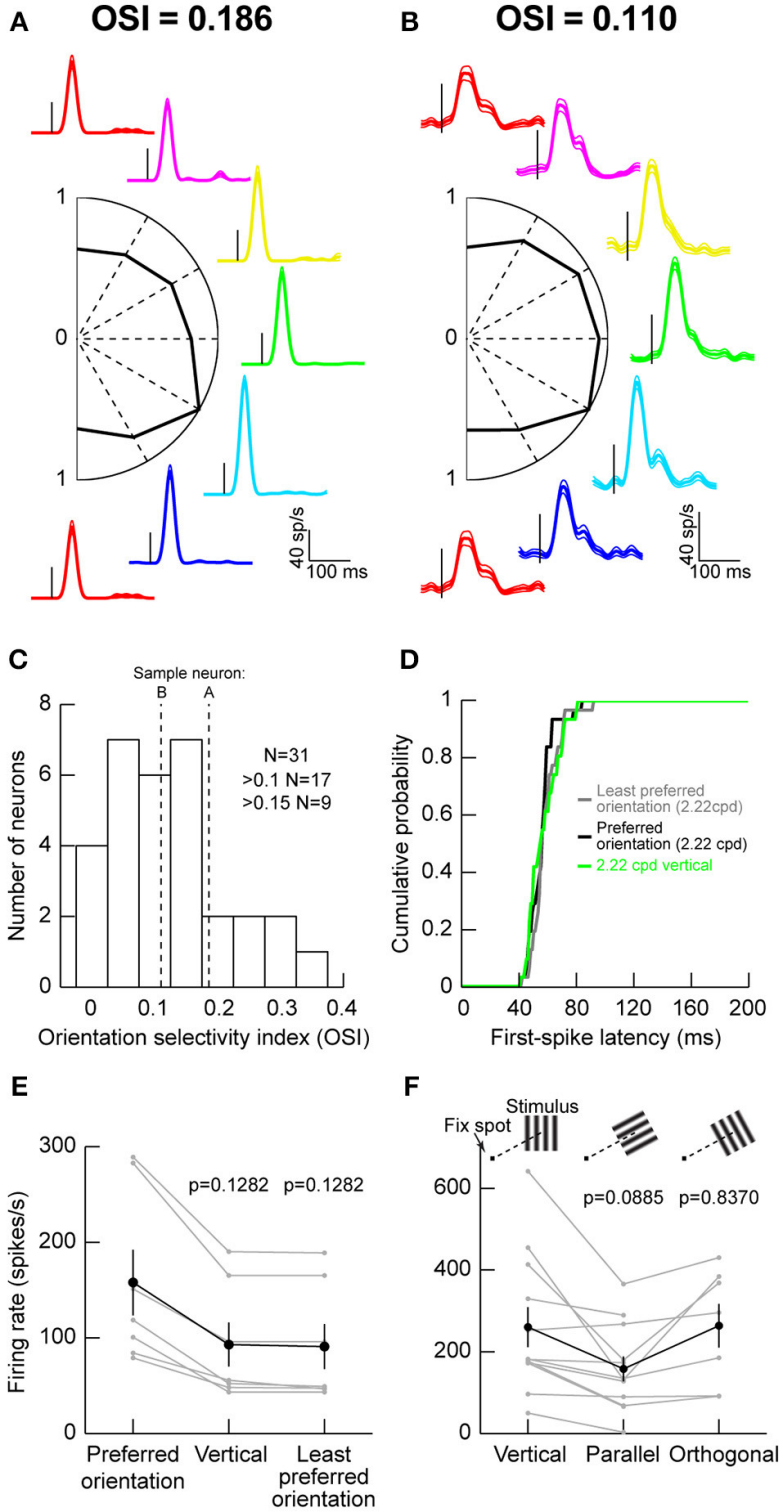
Orientation tuning in the macaque superior colliculus (SC). **(A)** Visual responses of an example SC neuron to gratings of different orientations (color-coded) presented within the neuron's visual response field (RF). The firing rate curves are graphically arranged according to the grating orientation. For example, the red curves are for vertical gratings, and the green curve is for horizontal gratings. Each curve is aligned on stimulus onset (small vertical black line in each firing rate curve), and the data for each orientation show the mean firing rate across trials, accompanied by s.e.m. curves as thin lines. The scale bars in the bottom right apply to all shown firing rate curves. In the center of the panel, we plotted the peak visual response (normalized to the maximum at one of the orientations) as a function of grating orientation. This neuron preferred an orientation of 30° below horizontal (cyan firing rate curve). The orientation selectivity index (OSI; Materials and Methods) of this neuron is indicated above the data. **(B)** A second neuron with weaker orientation selectivity. The neuron responded almost equally well to all orientations. **(C)** Distribution of OSI values across our population. The majority of neurons had mild orientation selectivity. **(D)** Consistent with this, first-spike latency did not depend strongly on stimulus orientation. The figure shows the distribution of first-spike latencies for the least preferred orientation, the most preferred orientation, or a vertical grating like that we used in Chen et al. (2015). In all cases, first-spike latency was similar (*p* = 0.5884, Kruskal-Wallis test). **(E)** Also, for neurons not preferring vertical orientations, the differences in visual responses between the most preferred, the least preferred, and the vertical orientations were mild. The black curve shows the summary across neurons, and the gray curves show responses for individual neurons. The *p*-values indicate the results of a Wilcoxon rank sum test comparing a given orientation (e.g., vertical or least preferred) to the preferred orientation of a given neuron. **(F)** Testing parallel vs. orthogonal orientations relative to the line connecting the fovea to the RF stimulus location (see schematics above the data points showing a fixation spot and a grating of a given orientation). Parallel gratings (oriented parallel to the line connecting the fovea to the neuron's RF hotspot) had weaker responses than orthogonal ones (oriented orthogonal to the line connecting the fovea to the neuron's RF hotspot). Error bars in all panels denote s.e.m.

We recorded visual responses in macaque monkeys that were fixating a small spot, while we presented an oriented grating of 2.22 cycles/° (cpd) within a neuron's visual response field (RF). The grating was stationary and was presented for ~200 ms. Figure 1A shows the responses of an example neuron exhibiting some orientation selectivity. Each colored curve shows raw firing rates from the neuron when a specific orientation was presented, and the orientations are arranged graphically according to the graphic placement of a firing rate plot (e.g., the magenta trace reflects responses to a grating that was tilted slightly rightward of purely vertical). The central part of the figure shows a plot of peak firing rate after stimulus onset as a function of grating orientation. As can be seen, this neuron responded the most for a grating oriented to the bottom right (the cyan trace). We computed an OSI according to the literature from early cortical visual areas (Hansel and van Vreeswijk, 2012) (Materials and Methods), and we found that this neuron had an OSI of 0.186. However, this neuron did not represent the majority of SC neurons that we recorded. Instead, we were more likely to encounter neurons like that shown in Figure 1B. In this case, orientation tuning was lower, as also reflected by the lower OSI value than in Figure 1A. Across the population, only approximately one third of our neurons had strong OSI values >0.15 and only approximately one half had mild OSI values >0.1 (Figure 1C).

We also checked the efficiency with which neurons responded to different orientations, by measuring first-spike latency as we had done earlier (Hafed and Chen, 2016). Regardless of whether a grating was vertical (Chen et al., 2015) the least preferred orientation of a given neuron, or the most preferred orientation of a given neuron, first-spike latency was similar (Figure 1D). This means that first-spike latency was similar across orientations.

Since in our previous studies, we used vertical gratings to study SC modulations around the time of microsaccades (Chen et al., 2015; Chen and Hafed, 2017), we also confirmed that this was a reasonable strategy for the study of SC neurons. For neurons not preferring vertical orientations, we plotted visual responses to either the most preferred, the least preferred, or a vertical orientation and compared them (Figure 1E). We found modest changes in responses across all of these conditions, suggesting that vertical gratings were still able to evoke responses in SC neurons. This is consistent with the results of Figures 1A–D, and consistent with our use of vertical gratings in recent studies (Chen et al., 2015; Chen and Hafed, 2017).

We also checked whether placing a grating orthogonal or parallel to the line connecting the fovea to the grating location (or, equivalently, RF location) matters. We collected data from 11 extra neurons, and we found that parallel gratings (Figure 1F) were least effective in driving SC neurons. Orthogonal gratings, on the other hand, resulted in stronger responses (*p* = 0.016, Wilcoxon signed rank test comparing orthogonal and parallel responses in the neurons with both conditions tested simultaneously). This suggests that, even though orientation selectivity in the primate SC is low in general, there is still substantial dependence of orientation sensitivity on the location of a given neuron within the SC topographic map. This is reminiscent of rodent observations of potential orientation columns in the SC independent of the basic spatial topography (Feinberg and Meister, 2015).

### Sharper contrast sensitivity curves for small stimuli in response fields

**Figure 2:**
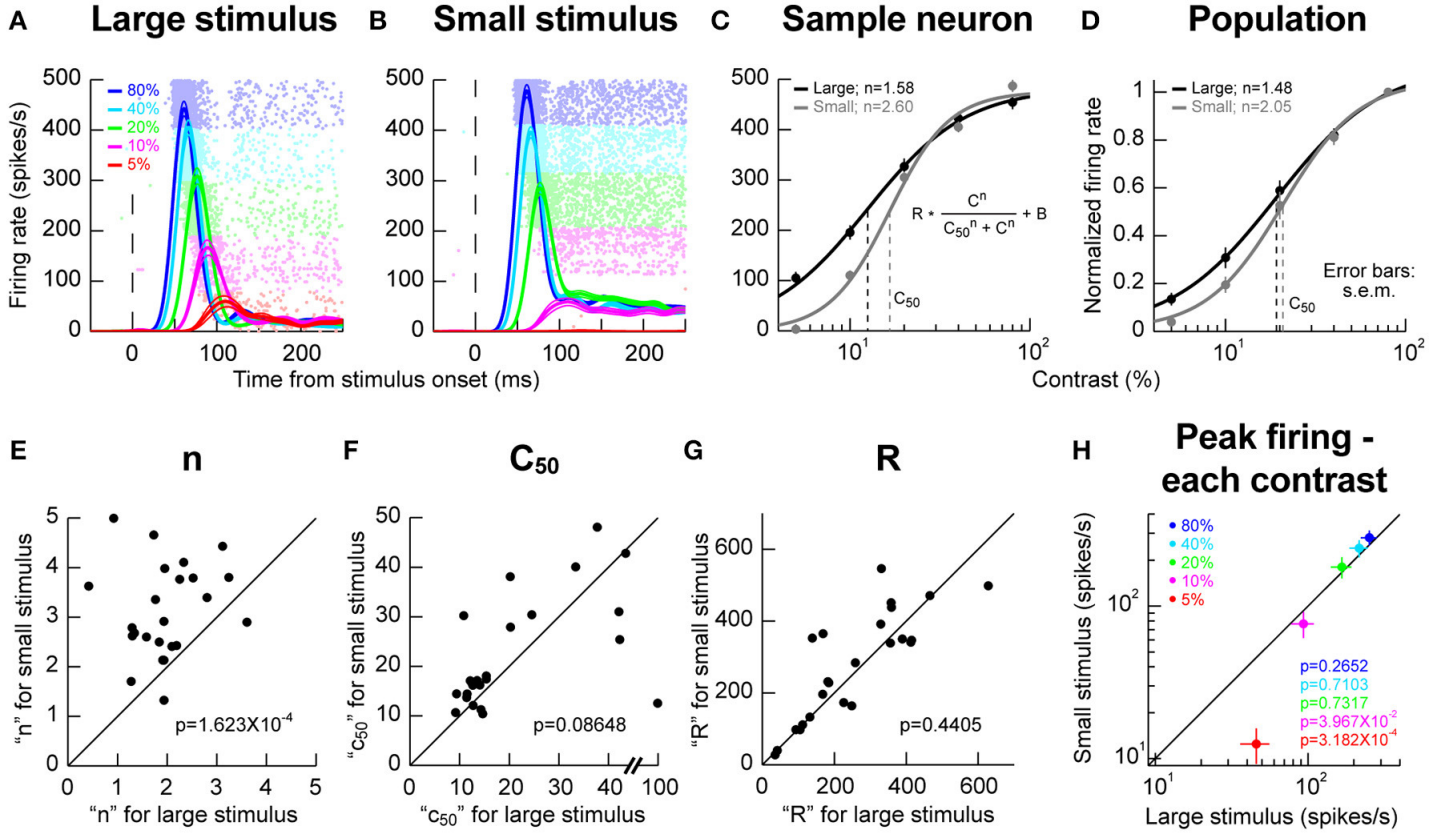
Stimulus size effects on contrast sensitivity. **(A,B)** Responses of a sample neuron to different stimulus contrasts (color-coded according to the legend). The RF hotspot eccentricity of the neuron was 5.6° from the center of gaze and in the upper visual field. On the left **(A)**, the stimulus filled the RF (2° in size); on the right **(B)**, the stimulus was much smaller (0.5° in size). The responses to each individual contrast are shown as an average firing rate curve surrounded by s.e.m. traces across trial repetitions. The faint dots behind the firing rate curves show the individual action potential rasters from each contrast, with each row indicating a trial repetition of a given contrast. The contrasts were interleaved in the experiments, but the rasters are grouped here to facilitate inspection of the results. The responses of the neuron to stimulus contrast were similar except that the evoked response at low contrasts (5 and 10%) was much weaker for the small stimulus than for the big stimulus. **(C)** We plotted the evoked responses (peak visual response 20–150 ms after stimulus onset) as a function of contrast, and we fitted the data with the shown equation. In this neuron, the slope parameter (*n*) was different for large and small stimuli. The semi-saturation contrast (c50) was also apparently altered. **(D)** However, across the population, the only significant effect was on n, meaning that contrast sensitivity curves were sharper for small stimuli. **(E–G)** The parameters of the contrast sensitivity curve equation (shown in C) across neurons for small vs. large stimuli. Across the population, only the parameter n showed a significant effect of stimulus size. The shown *p*-values indicate the result of a Wilcoxon signed rank test comparing small and big stimuli. **(H)** Consistent with this, when we plotted peak firing rate as a function of contrast, we found that the rate was significantly lower for small vs. big stimuli (*p*-values in the colored-text; Wilcoxon signed rank test comparing small and big stimuli) only for low contrasts. For high contrasts, neural sensitivity was similar for different stimulus sizes. Error bars in all panels denote s.e.m.

We next explored potential local RF interactions by changing stimulus size at a given location. We ran conditions using a vertical grating as in Chen et al. (2015), but this time, we compared responses when the grating was either filling as much of the RF as possible or when the stimulus was significantly smaller (Materials and Methods). We varied the contrast of the grating from trial to trial in order to obtain contrast sensitivity curves. In the sample neuron of Figures 2A,B, we obtained an expected dependence of visual response strength on stimulus contrast; the higher the contrast, the higher and earlier the visual response was, and this happened for both a large and a small grating in the RF. However, closer inspection of the contrast sensitivity curves revealed an additional property: there was a sharpening of these curves for the smaller stimulus (Figure 2C), primarily because of a change in response strength for low contrast stimuli. In this sample neuron, semi-saturation contrast (c50) appeared to be also affected, but this effect was not significant across the population (Figure 2D). Instead, across the population, the only significant effect was on the slope (*n*) of the contrast sensitivity curve. We confirmed this by plotting in Figures 2E–G the different parameters of the contrast sensitivity curve equation (displayed in the inset of Figure 2C) for each neuron. Across the population, only the slope parameter of contrast sensitivity curves was significantly different between small and large stimuli (*p* = 1.623 × 10^−4^, Wilcoxon signed rank test). This effect is also evident in Figure 2H, in which we plotted the peak firing rate for each stimulus contrast for either small or large stimuli. Firing rates between small and large stimuli were similar for high contrasts regardless of stimulus size. However, for lower contrasts (5 and 10%), responses were weaker in the smaller stimuli than in the larger stimuli, again consistent with a sharpening of contrast sensitivity curves. We should also note here that for these population analyses, we accepted all neurons passing our explained variance tests for the curve fitting procedures (Materials and Methods). For one of these neurons, a c50 of 100% was observed (Figure 2F), which constituted an outlier when compared to the rest of the population for assessments of c50 effects. Removing this outlier resulted in significant c50 modulations across the population in Figure 2F (*p* = 0.0029, Wilcoxon signed rank test). This effect on c50 was still nonetheless consistent with our observation that the effect of stimulus size primarily acted through a change in SC response strengths for low contrast stimuli.

### Higher sustained activity for small stimuli in response fields

The results above with small stimuli might suggest differences in local lateral interactions caused by an extended stimulus (Humphrey, 1968; Schiller and Koerner, 1971; Cynader and Berman, 1972; Goldberg and Wurtz, 1972; Updyke, 1974; Marrocco and Li, 1977). This idea was rendered clearer when inspecting sustained firing rates after the initial onset transient. In the sample neuron of Figures 2A,B, it can be seen that sustained activity was elevated for the small stimuli, even when the evoked response was itself weak. For example, for 10% stimulus contrast, even though the initial evoked response was much weaker in the small stimulus configuration than in the large stimulus configuration, the sustained activity was significantly higher in the small stimulus configuration than in the large stimulus configuration (*p* = 0.0011, two-sample *t*-test). This suggests that with the large stimulus, an inhibitory effect kicked in after the initial excitatory stimulus transient, even though the stimulus was contained within the classical RF boundaries and not extending outside as might be expected from surround suppression.

**Figure 3:**
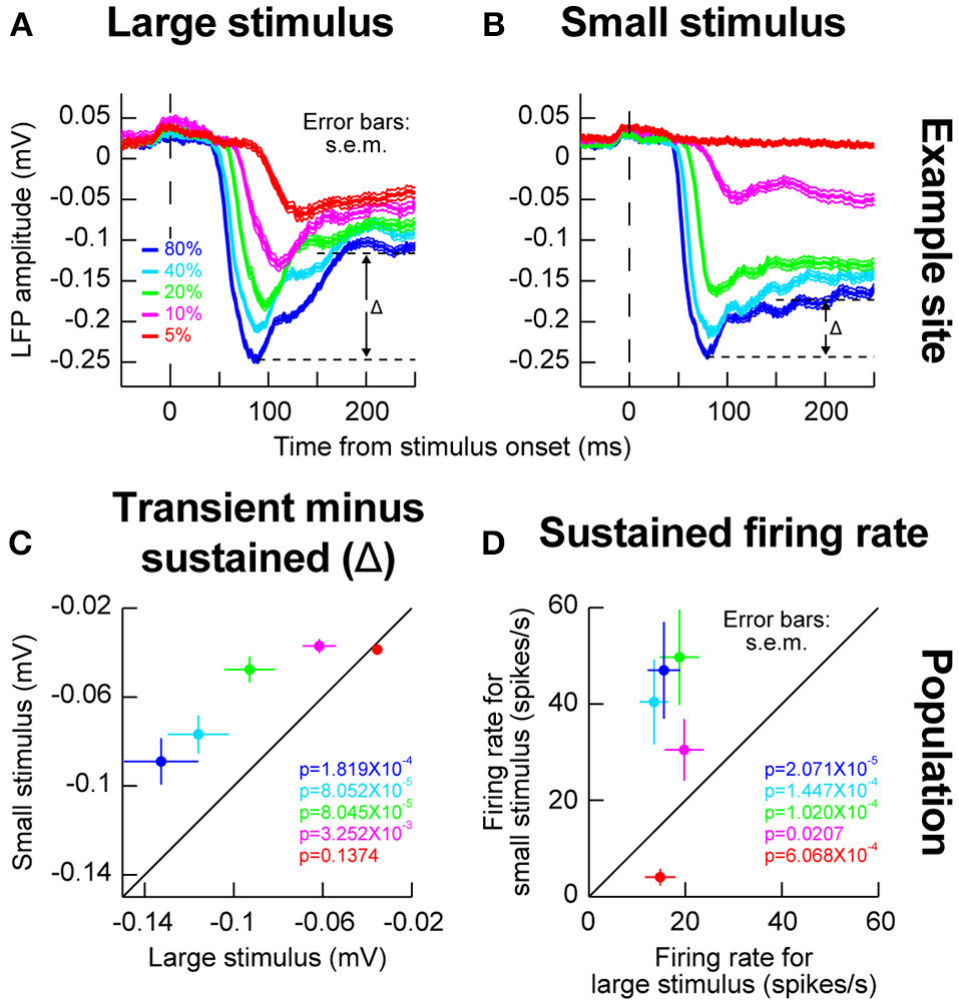
Local population activity for different stimulus sizes within a given RF. **(A)** Local field potential (LFP) responses for the same electrode track as the one for which the example neuron of Figure 2 was isolated. For the large stimulus, evoked LFP response (a negative-going deflection) resembled the expectations based on firing rate analyses in Figure 2. **(B)** When the stimulus was small, the evoked response at 10 and 5% contrast was much smaller (compare magenta and red curves between this panel and **A)**. However, note that the sustained response component remained elevated (i.e., remained more negative long after the evoked response had subsided); the difference between the peak evoked response and the transient response (Δ) was smaller for the small stimulus than for the big stimulus (compare Δ in this panel to **A)**. This means that in the big stimulus, after the evoked excitatory response, a potential inhibitory signal started to kick in to suppress neural activity, explaining the higher sustained firing rates for small stimuli in Figures 2A,B even at low stimulus contrasts (10%). **(C)** Across electrode sites, the value of Δ was bigger for the big stimulus than for the small stimulus across contrasts (except for the lowest contrast which already evoked very weak responses). **(D)** Consistent with this, across neurons, sustained firing rates for small stimuli were consistently higher than sustained firing rates for big stimuli, even for low 10% contrasts for which the initial evoked response was much weaker (Figure 2). Again, for the lowest contrast, the effect is not present but this contrast evoked very weak or non-existent responses from most neurons to begin with. Thus, stimulus onset in the SC is dominated by an early excitatory drive followed by subsequent inhibition even within a classical RF boundary. Error bars in all panels denote s.e.m., and the shown *p*-values indicate results of a Wilcoxon signed rank test comparing small and big stimuli.

We also wanted to investigate whether the local population activity around our recording electrodes behaved similarly in this regard to our isolated single neurons or not (Zhang and Li, 2013). We therefore next analyzed LFP's, and we still observed evidence for such an inhibitory effect. For example, in Figures 3A,B, we plotted LFP evoked responses for different contrasts and different stimulus sizes from the same electrode penetration in which the sample neuron of Figures 2A,B was isolated. As can be seen, the sharpening of contrast sensitivity curves can be clearly seen in the LFP evoked responses. For example, for the small stimulus, the initial evoked LFP transient for 10% contrast was much weaker than with a large stimulus (compare the magenta curves of Figures 3A,B). On the other hand, the initial evoked responses for high contrast stimuli were very similar whether the stimuli were large or small. Interestingly, after the initial evoked transient had subsided, there was a bigger change in LFP amplitude between the transient and sustained response (labeled Δ in Figures 3A,B) for large stimuli than for small stimuli, suggesting a potential inhibitory effect kicking in after the initial excitation. Across the population of experiments, this effect of a bigger Δ with the big stimuli persisted (Figure 3C), and it was mirrored by higher sustained firing rates for small stimuli in the isolated neurons (Figure 3D). Naturally, these effects were weakest for the lowest stimulus contrast (5%), because this contrast evoked the weakest responses anyway (Chen et al., 2015).

Therefore, our experiments with small and large stimuli revealed potential local lateral interactions in and around classical SC RF's. These interactions are not identical to inhibitory surround interactions in V1 (Vaiceliunaite et al., 2013). For example, in our case, the bigger stimulus did not reduce the SC response for high contrast stimuli as might be expected from surround suppression in V1 (Vaiceliunaite et al., 2013) suggesting that this stimulus was still within the classical RF boundaries in our experiments. Nonetheless, subsequent inhibition in neural responses during the sustained stimulus interval still occurred in our neurons.

### Changes in first-spike latency for small stimuli in response fields

**Figure 4:**
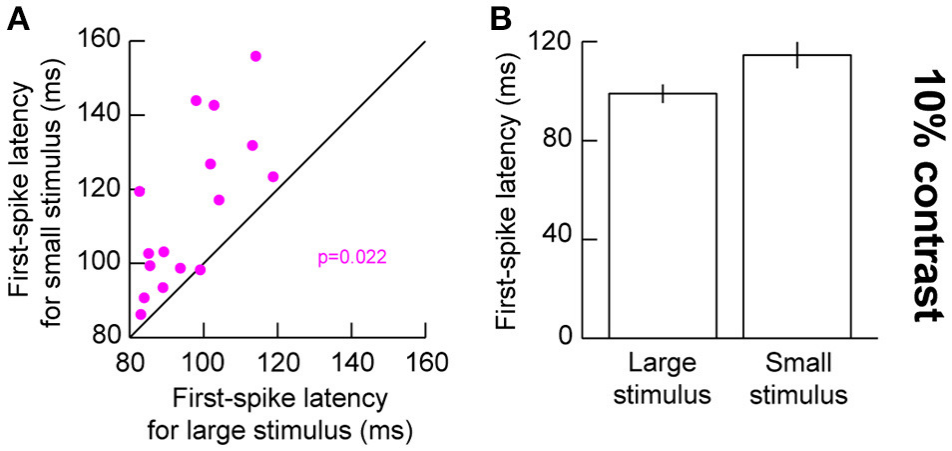
Visual response latency for low contrast large and small stimuli. **(A)** First-spike latency for neurons when a 10% contrast grating was presented in their visual RF's. The x-axis shows first-spike latency with a large stimulus in the RF, and the y-axis shows first-spike latency with a small stimulus in the RF. **(B)** Summary of first-spike latency across our population as a function of stimulus size. The small stimulus was associated with a longer latency of visual responses. Error bars denote s.e.m.

The above results suggest that for small stimuli, low contrast stimuli evoke weaker responses. This should also result in later evoked responses, since visual response latency tends to be correlated with visual response amplitude in the SC (Marino et al., 2012; Chen and Hafed, 2017). We confirmed this to be the case. We plotted first-spike latency as we had done earlier (Hafed and Chen, 2016), but now relating it to stimulus contrast. For 10% contrast in the presented gratings, our earlier results above (Figure 2) showed that visual responses were significantly different for small and big stimuli. We thus compared first-spike latencies at 10% contrast between the different stimulus sizes (Figure 4). For the small stimulus, first-spike latency at 10% contrast was significantly longer than for the big stimulus (*p* = 0.022, Wilcoxon rank sum test) consistent with the sharper decrease in response gain for this contrast with small stimuli (Figure 4). This effect was not significant for the higher stimulus contrasts (*p* = 0.277, 0.473, 0.787, and 0.600 for 5, 20, 40, and 80% contrast, respectively, Wilcoxon rank sum test), again consistent with our interpretation above (Figure 2) that stimulus size altered the slope of the contrast sensitivity curve of SC neurons without altering other parameters like semi-saturation contrast or maximal firing rate. It should be noted here that we used a stricter unpaired statistical test in this analysis, as opposed to a paired Wilcoxon signed rank test, because not all neurons in our population exhibited responses in both small and large stimuli (especially for low contrast stimuli). Therefore, we could not estimate first-spike latency in such cases (assigning an arbitrary value of infinity for no spiking responses might have biased our measurements unnecessarily).

### Preference for primarily 10–20 Hz temporal frequency by primate SC neurons

**Figure 5:**
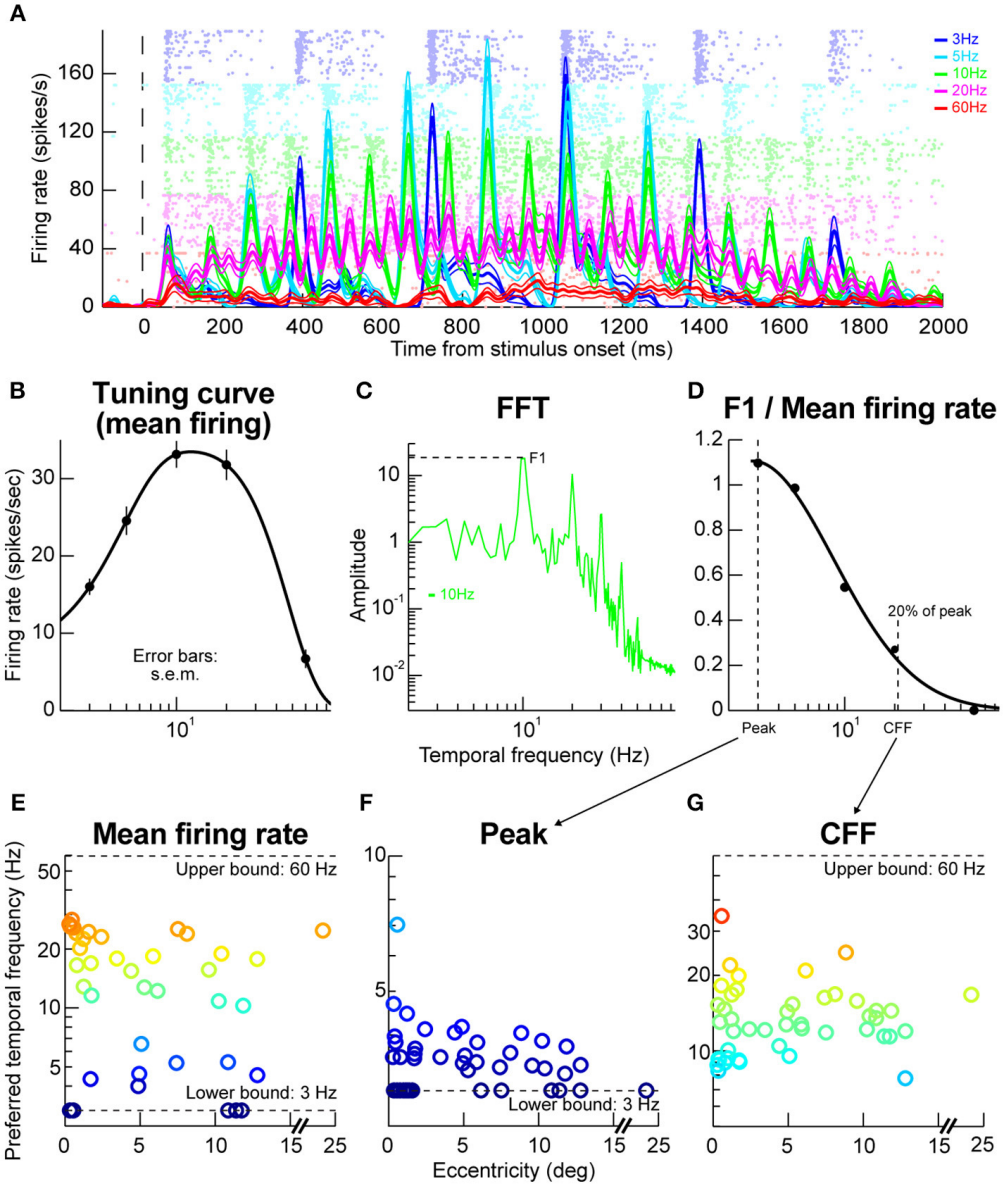
Temporal frequency tuning by macaque SC. **(A)** Example firing rates and raster plots from a neuron exposed to flicker of different frequencies. At low frequencies, the neuron emitted phasic responses to individual stimulus events (e.g., at 3 Hz; blue curves and rasters). At higher frequencies, the individual phasic events started to merge or fuse (e.g., at 20 Hz; magenta curves and rasters). At even higher frequencies (e.g., 60 Hz; red curves and rasters), the neuron stopped responding completely even though the stimulus was practically almost permanently on the display. The formatting of this panel is similar to that described for Figures 2A,B above (i.e., for the faint dots describing action potentials and firing rate curves describing averages and s.e.m. boundaries), and error bars denote s.e.m. **(B)** The tuning curve of the neuron obtained by plotting mean firing rate as a function of temporal frequency. The neuron responded best for 10–20 Hz frequencies. **(C)** Fourier transform of firing rate for the same neuron with 10 Hz stimulus flicker. The neuron had a dominant harmonic at 10 Hz (F1) along with power at different frequencies (e.g., multiples of 10 Hz), including also DC (0 Hz), indicating a non-zero average response (sometimes referred to as F0). **(D)** We plotted the power at F1 divided by the mean response for this condition (or the DC response), to estimate how big the phasic response to individual stimulus events was relative to the overall average. For this same neuron, the phasic response at 3 Hz was very strong (also evident in **A)**. At near 20 Hz, the individual phasic responses (aligned to stimulus flicker) were much weaker compared to the overall average firing rate, suggesting “flicker fusion.” We defined the critical flicker fusion frequency as the frequency for which F1/F0 was 20% of the peak. **(E)** Preferred temporal flicker frequencies of all neurons based on tuning curves like in **(B)**, and plotted as a function of each neuron's preferred retinotopic eccentricity. There was no apparent eccentricity dependence of temporal tuning. **(F,G)** the parameters of the curve in **(D)** across all our neurons. Most neurons had a critical flicker fusion frequency near 20 Hz. Error bars in all panels indicate s.e.m. Also, the colors in **(E–G)** are provided as a visual aid, and they go from cool to warm with increasing y-axis values.

We next explored the temporal properties of SC neurons. We presented flickering gratings and varied the flicker frequency from trial to trial (Materials and Methods). The contrast of the gratings was gradually increased and then gradually decreased to minimize onset transients, so it was expected that neural responses would be time varying in this experiment; the logic of the experiment here was that the time variation with stimulus contrast would be also accompanied by time variation associated with the stimulus flicker (Materials and Methods). Figure 5A shows example results that we obtained from a sample neuron. At low flicker frequencies (e.g., 3 Hz), the neuron responded in a phasic manner to each stimulus onset event (in individual flicker cycles) and the phasic response reflected the gradual increases and decreases in stimulus contrast near the beginning and end, respectively, of a given trial (Materials and Methods). At higher frequencies (e.g., 20 Hz), the phasic events were less obvious than for low frequencies (e.g., 3 Hz), and they were replaced with a more sustained response. This means that 20 Hz was close to the critical flicker fusion frequency (Wells et al., 2001) of this neuron. Interestingly, increasing the frequency more to 60 Hz, resulted in a much weaker neural response even though the stimulus was practically constantly presented on the monitor for the entire duration of the trial. This means that the neuron exhibited tuning for temporal frequencies, and that 60 Hz was outside the neuron's preferred frequency range.

We assessed the sample neuron's frequency “tuning curve” as done in the previous literature for cortical visual neurons, for example in rats (Wells et al., 2001), by measuring the average firing rate during trials and plotting it as a function of stimulus flicker frequency. The logic of such tuning curves is similar to the logic of obtaining spatial frequency tuning curves as a function of grating spatial frequency, for example in our macaque SC neurons (Hafed and Chen, 2016). Figure 5B shows the temporal frequency tuning curve obtained for the same sample neuron as in Figure 5A, and the curve was obtained by fitting the data to a difference-of-Gaussians equation (Materials and Methods). As can be seen, the neuron was most sensitive to temporal frequencies of 10–20 Hz. We also assessed the amplitude of the neuron's sensitivity to a given frequency. As others have done for cortical visual neurons (Wells et al., 2001), we computed a so-called F1/F0 ratio. Briefly, we performed a Fourier transformation of firing rate observed when a given temporal flicker frequency was presented to the neuron (e.g., 10 Hz in Figure 5C). As expected, we obtained a primary harmonic at the stimulus frequency (F1; 10 Hz in Figure 5C). The amplitude of the harmonic was then divided by the mean firing rate (or the 0 Hz response of the neuron), and we repeated this for different stimulus frequencies that were presented. For the neuron in Figure 5A, this procedure resulted in the F1/F0 curve shown in Figure 5D. This curve means that the phasic response at 3 Hz was much stronger relative to the mean firing rate than, say, the phasic response at 10 Hz, which is also evident from inspecting Figure 5A. Additionally, this curve was used to define the flicker fusion frequency or the frequency at which the phasic response at the stimulus frequency was much reduced (to 20% of the peak). In the neuron of Figure 5D, this frequency was just above 20 Hz, again consistent with the raw data in Figure 5A. In other words, above ~20 Hz, the neuron just emitted a more-or-less constant response as opposed to a phasic response to each stimulus cycle.

Across the population, our neurons temporal frequency tuning curves were relatively broad (Figure 5E), with a range of neurons preferring a range of flicker frequencies, but none of the neurons preferred >30 Hz. Similarly, most neurons had a critical flicker fusion frequency of ~20 Hz (Figures 5F,G). These temporal SC properties are very similar to those observed in the visual capabilities of blindsight patients who lose portions of their primary visual cortex (Trevethan and Sahraie, 2003).

## Discussion

In this study, we explored visual properties of the rhesus macaque SC, as a continuation of our recent efforts in this regard (Chen et al., 2015, in press; Hafed and Chen, 2016; Chen and Hafed, 2017; Veale et al., 2017; Yoshida et al., 2017). We found that primate SC neurons exhibit mild orientation selectivity. We also found that within the boundaries of a classical RF of an SC neuron, increasing stimulus size can increase the size of the initial volley of stimulus-evoked action potential rates, but it also increases subsequent inhibition. The net result is that bigger stimuli can evoke stronger initial responses but weaker sustained responses. Finally, we explored the primate SC's temporal frequency tuning properties, and we found flicker fusion rates of ~20 Hz. Thus, the SC's temporal response profile lies in an intermediate range between the slow rod-mediated component of vision at the level of the retina and the faster cone-mediated component.

Our results on orientation tuning are related to recent rodent SC work. For example, it was suggested that the mouse SC contains orientation columns (Feinberg and Meister, 2015), much like primate primarily visual cortex contains such columns. In other words, according to these authors, different regions of the SC topographic map might over-represent particular orientations, such that there is an additional “feature map” for orientation in the SC, in addition to this structure's topographic representation of space. Our results so far cannot fully address whether the primate SC would also contain such “feature maps” in addition to spatial topography. The clearest evidence that we have in our data for such potential feature maps may relate to Figure 1F, in which we observed that orthogonal gratings (relative to the line connecting the fovea to the RF location) evoked stronger visual responses than parallel ones. This suggests that orientation tuning properties in the primate SC can depend on a given neuron's RF location within the SC topographic map, and this is an interesting question that needs to be further investigated with significantly larger data sets. There was apparently also no indication of such feature mapping in recent marmoset monkey SC work (Tailby et al., 2012). In any case, given ecologically-driven large asymmetries in representing the upper and lower visual fields (Hafed and Chen, 2016), it is certainly conceivable that similar ecological constraints may indeed result in over-representation of certain orientation preferences in different parts of the primate SC's representation of visual field locations. Such asymmetries would most likely exist if they serve orienting behavior with rapid gaze shifts, since the primate SC is particularly relevant for such gaze shifts.

We also explored potential lateral interactions in and around RF's, and we explored temporal tuning properties. We believe that such experiments and results should be followed up on in subsequent studies, especially if one were to understand the broader contributions of the SC to active perception. For example, eye movements under natural conditions might be associated with different peri-saccadic perceptual phenomena depending on the prevalent scene statistics in natural images (Burr et al., 1994), and there is evidence that the SC plays a critical role in these perceptual phenomena (Chen and Hafed, 2017; Chen et al., in press). Given that individual cell types (like purely visual vs. visual-motor neurons) might be particularly dissociated in these kinds of phenomena (Chen and Hafed, 2017) even though it is a visual response that is ultimately modulated in all cases, there is a need to dissect the circuit mechanisms of the primate SC in much more detail, and specifically in terms of visual properties. Similarly, the role of the SC in target selection and attention (Li and Basso, 2005; Krauzlis et al., 2013; Basso and May, 2017; Crapse et al., 2018; Odegaard et al., 2018) would be even better understood than already present today if phenomena like lateral interactions and temporal response profiles were fully characterized. Finally, on top of all of the above, there still remains a distinct possibility that the SC contributes to coarse initial visual analysis of scenes, particularly to allow for efficient approach or evade responses by the organism. Indeed, combined with our earlier work, like (Hafed and Chen, 2016) and (Chen et al., in press) as well as other work, like (Nguyen et al., 2014), there is evidence that the visual properties of the SC may be relevant for behavior that is based on (at least coarse) scene understanding. We find this possibility intriguing and worthwhile for future research endeavors.

We also find our results intriguing because of how they may link to the role of the SC as a potential alternative visual pathway from the primary geniculo-cortical pathway that is used by primates. In particular, blindsight is a phenomenon that happens after lesion of the primary visual cortex, and it is characterized by an ability of patients, with very little or sometimes no awareness of a stimulus presented in the blind field, to perform discrimination tasks above chance level, especially if the stimulus is salient (Weiskrantz et al., 1974; Cowey and Stoerig, 1991; Ptito and Leh, 2007; Cowey, 2010; Leopold, 2012). The visual stimuli that are optimal for these patients are critical. Specifically, these patients perform the best with first-order low spatial frequency patches, with a cut off of around 3 cpd (Sahraie et al., 2002, 2010; Trevethan and Sahraie, 2003). Transient stimuli are usually better, with a range around 10–33 Hz, peaking at around 20 Hz. These tuning properties are very similar to what we found in our SC neurons, both in this study (for temporal frequency) and in earlier work (for spatial frequency) (Chen et al., in press). Patients can also perform color discrimination tasks (Boyer et al., 2005; Silvanto et al., 2008). It is also known that the pupillary reflex can be a reliable predictor of performance (Sahraie et al., 2002). Because the lateral geniculate nucleus (LGN) and pulvinar project directly to extrastriate cortex, and because both of them also receive superficial SC and retinal input, it could be that blindsight reflects residual vision from this alternative visual pathway through LGN, SC, or pulvinar, or all of them to the extrastriate cortex (Cowey and Stoerig, 1991; Isa and Yoshida, 2009; Leopold, 2012). The remarkable observation based on our results is that the SC visual properties are quite similar to those of blindsight patients, which could add to the discussion on whether a collicular pathway is more or less important during blindsight than the other potential pathways.

## Author contributions

C-YC and ZH designed the experiments, collected and analyzed the data, and wrote the manuscript.

### Conflict of interest statement

The authors declare that the research was conducted in the absence of any commercial or financial relationships that could be construed as a potential conflict of interest.
